# Endoscopic Resection of a Large Colonic Lipoma Simulating a Tumor Revealed by Hemorrhage: A Case Report and Literature Review

**DOI:** 10.7759/cureus.24987

**Published:** 2022-05-14

**Authors:** Fatima Belabbes, Leila Abdallaoui Maane, Abderahmane Al Bouzidi, Abdennaceur El Idrissi Lamghari, Fedoua Rouibaa

**Affiliations:** 1 Gastroenterology and Proctology, Faculty of Medicine, Mohammed VI University of Health Sciences (UM6SS), Casablanca, MAR; 2 Oncology, Faculty of Medicine, Mohammed VI University of Health Sciences (UM6SS), Casablanca, MAR; 3 Pathology, Faculty of Medicine, Mohammed VI University of Health Sciences (UM6SS), Casablanca, MAR

**Keywords:** tumor, lipoma, endoscopic resection, colonoscopy, colon

## Abstract

Lipomas of the colon are benign tumors of the digestive tract. They are usually asymptomatic, and often discovered incidentally during a colonoscopy. However, lipomas larger than 2 cm may present with abdominal pain, bowel changes, and rectal bleeding. They may mimic cancer, depending on multiple factors including tumor size, location, and complications, which often makes preoperative diagnosis difficult. In this report, we discuss the case of a 34-year-old woman who presented with paroxysmal abdominal pain in the left iliac fossa withmoderate hematochezia that had been* *evolving for six months. The patient denied* *melena or hematemesis, and she had no significant medical history. Colonoscopy revealed a large polyp of over 5 cm located 40 cm from the anal margin. She underwent endoscopic resection without complications. The histological examination confirmed the lipomatous nature.

An accurate preoperative diagnosis of lipomas is necessary.It can often be difficult to choose between endoscopic and surgical treatment. The choice of treatment depends on the size and location of the tumor and complications. Endoscopic resection may obviate the need for surgery and can potentially reduce surgical morbidity. We aimed to report and discuss the management of this patient who underwent endoscopic resection for a large mass with a definitive pathology of colonic lipoma.

## Introduction

Lipomas of the colon are benign tumors of the digestive tract. Although most of them are asymptomatic, colon lipomas can present with symptoms such as pain, diarrhea, obstruction, and bleeding. Although it is a benign, nonepithelial tumor, the complication of bowel obstruction can be life-threatening [1]. Colonic lipomas can present challenges in the preoperative differential diagnosis between malignant and benign colonic neoplasms, which is the main problem associated with lipomas [2].

Endoscopic resection of large colorectal polyps has become the treatment of choice. However, surgery may still be performed frequently for large colorectal polyps that can be resected endoscopically. We report a case of large colonic lipoma with the endoscopic appearance of neoplastic polyp on colonoscopy that was removed with endoscopic resection. Through our observations and a review of the literature, we highlight the benefits of endoscopic resection for large polyps to avoid the cost and complications of surgical resections.

## Case presentation

A 34-year-old female patient with no specific medical or surgical history presented with paroxysmal abdominal pain with moderate hematochezia six months ago. The pain commonly radiated to the left iliac fossa. The patient reported no melena, hematemesis, or other extra-intestinal symptoms. She did not have anorexia, weight loss, or asthenia. The physical examination at admission found tachycardia with a heart rate of 80 beats per minute, a normal blood pressure, oxygen saturation of 94%, and discolored conjunctiva. An abdominal examination revealed no abdominal mass, and the digital rectal examination was normal.

**Figure 1:**
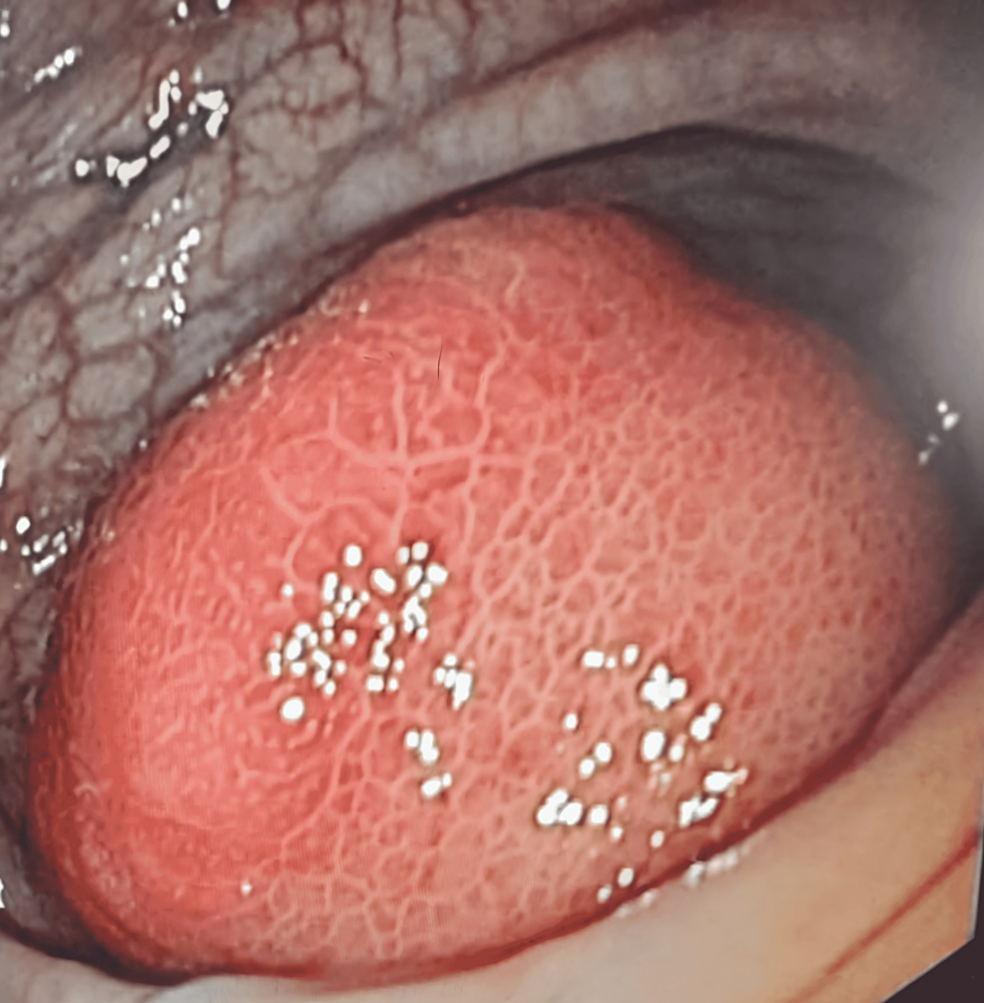
Colonoscopic image showing the broad-attachment colonic polypoid mass without ulceration (before endoscopic resection)

The results of the laboratory investigation were normal. Colonoscopy revealed a large mass (>5 cm in diameter) located 40 cm from the anal margin. It was round, polylobed, and mobile. Its base of implantation was difficult to visualize. The overlying mucosa was not ulcerated. The lesion was found to be a mass of hard consistency, which may lead to an impression of malignancy. It did not have the “classic” endoscopic features of colonic lipomas, such as the “tent sign” (Figure 1).

**Figure 2:**
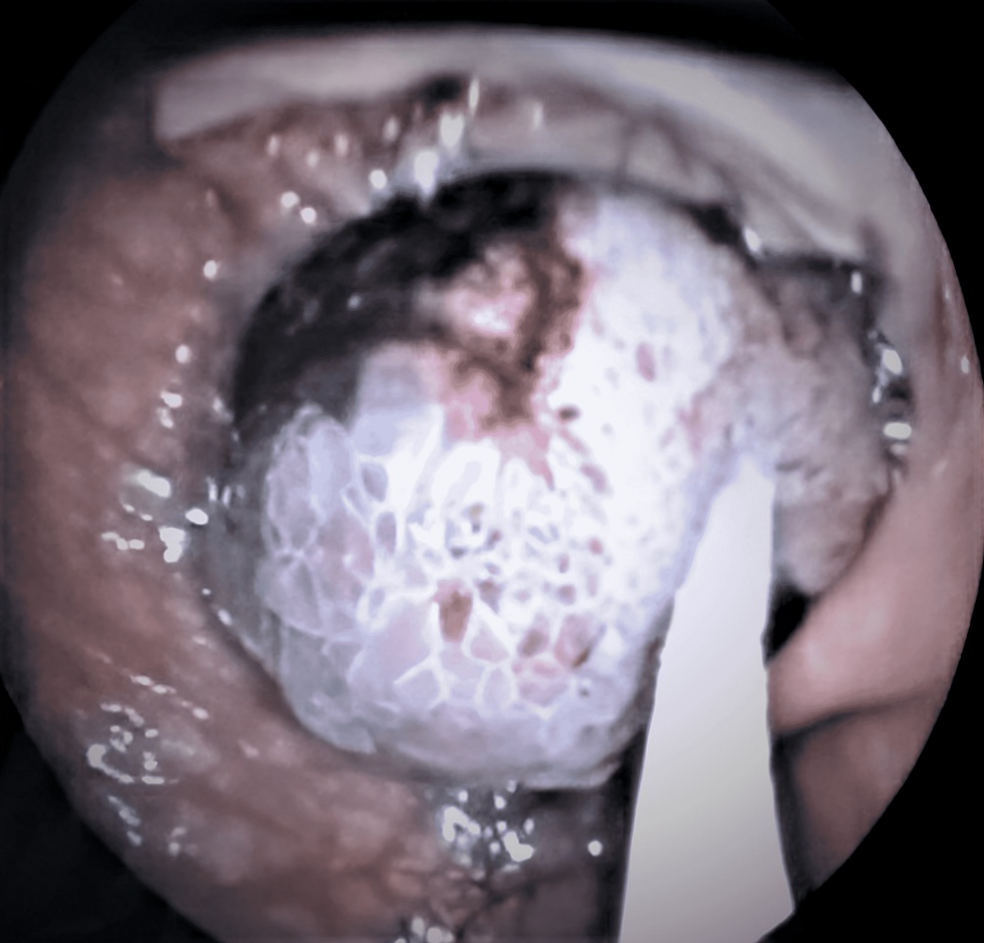
Hot snare endoscopic resection of the colonic mass

**Figure 3:**
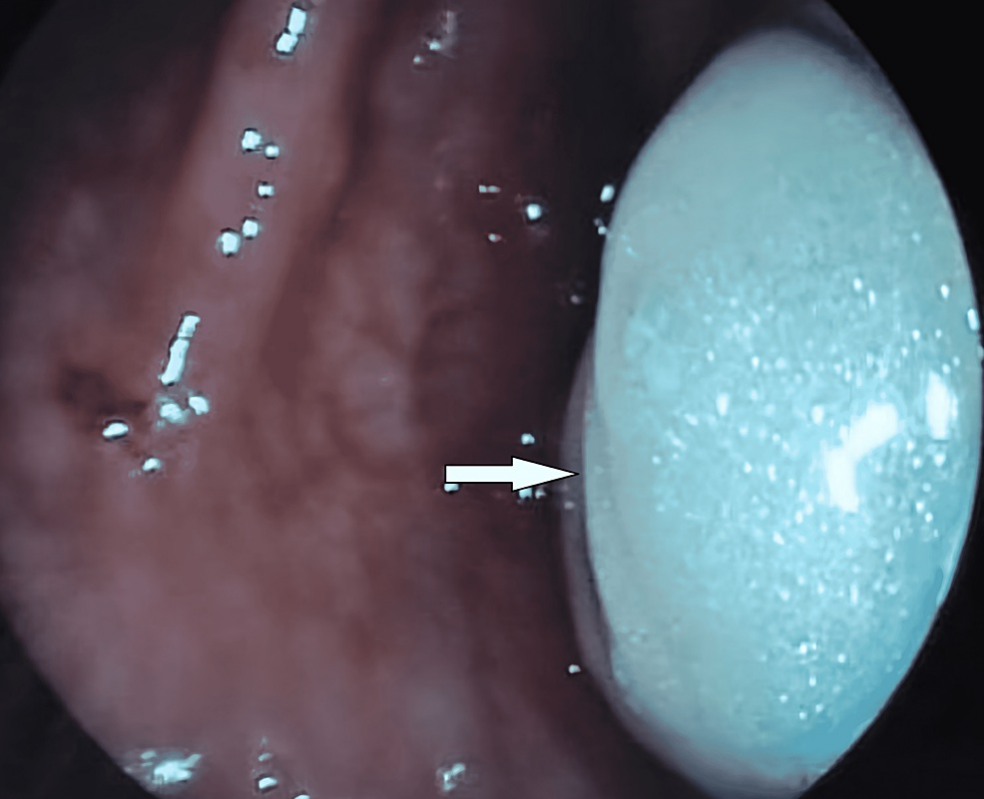
Post-resection colonoscopy showing the polypectomy site (white arrow)

The patient was informed about the increased risk of perforation and bleeding during endoscopic resection. The polyp was resected endoscopically by hot-loop diathermy after the injection of serum and adrenaline (Figure 2). The patient tolerated the procedure without any sequelae. There was no bleeding from the polypectomy site (Figure 3).

**Figure 4:**
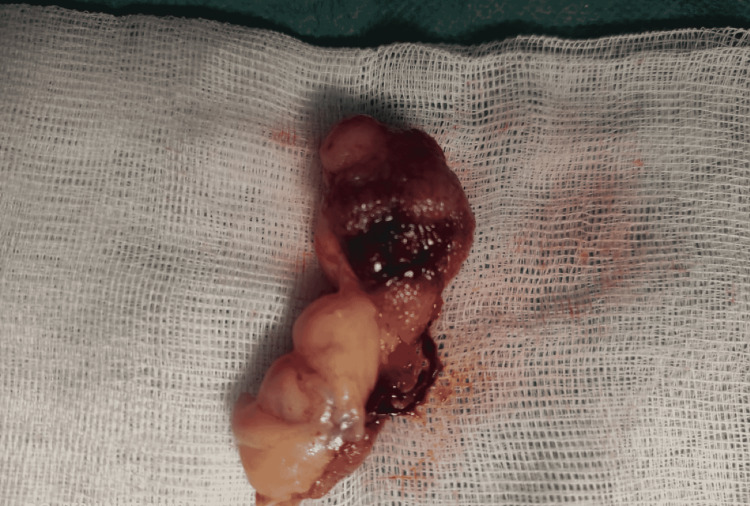
Large colonic lipoma after endoscopic resection

**Figure 5:**
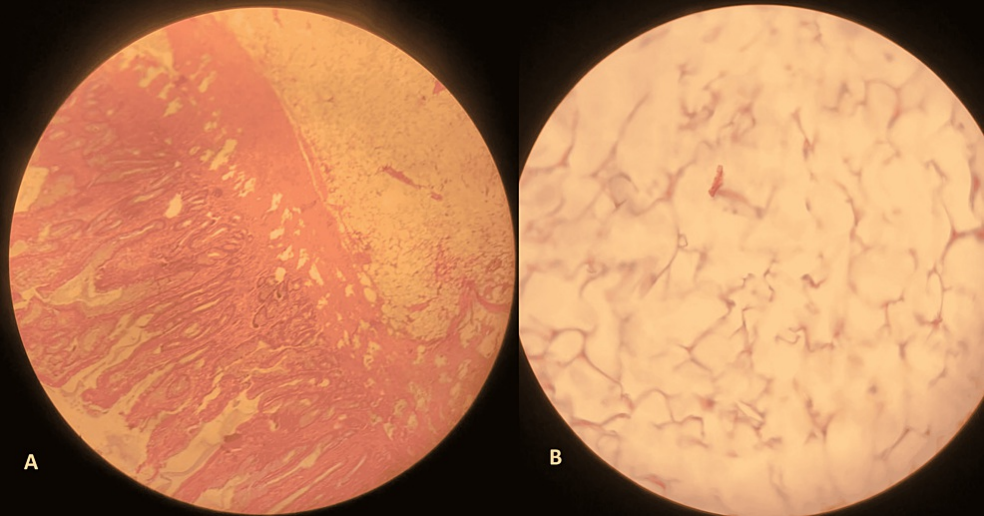
Pathological examination of the resected lesion The examination revealed (A) the proliferation of mature adipocytes in the colonic wall (HE, Gx40) and (B) adipocytes without cytonuclear atypia (HE, Gx100) HE: hematoxylin and eosin stain; G: grossissement

The polyp was then retrieved. The tumor was of hard consistency with submucosal location and did not show mucosal ulceration (Figure 4). The histological examination showed a morphological appearance compatible with a lipomatous polyp without any evidence of malignancy (Figure 5).

The evolution was marked by the disappearance of digestive symptomatology.

## Discussion

Lipomas of the gastrointestinal tract are rare entities and were first recognized by Bauer in 1757. They are more common in women than in men [3]. The overall incidence of this lesion is estimated to be 0.26% and represents 1.8% of all benign colonic lesions [4]. The colon is the most frequently affected segment, accounting for 65-75% of lipomas. The discovery of a lipoma is most often incidental during a screening colonoscopy or on a colectomy specimen [5].

The symptomatology is non-specific, essentially consisting of abdominal pain, constipation, and/or rectal discharge [1]. This symptomatology arises when the lesions exceed 2 cm in diameter. Intestinal intussusception is the most frequent complication of colonic lipomas [6]. In our case, the reason for consultation was hematochezia.

Colonoscopy is commonly used to diagnose lipomatous lesions. It is manifested by a swelling of the mucosa overlying the lipoma (tent sign), or by a soft mass under the biopsy forceps (pillow sign) [5]. Lipoma biopsy has no role in the diagnosis. Therefore, colonoscopy is a reliable method for the diagnosis of a typical lipoma. However, it may prove to be of no help when the lesion is atypical. Endoscopic ultrasonography (EUS) can also be used to clarify the nature of the mass. With regard to symptoms and endoscopic appearance, lipomas and tumors can be indistinguishable from each other. Even with abdominal imaging and direct colonoscopic visualization, lipomas may mimic neoplasms [4].

Large colonic lipomas continue to present challenges in the preoperative differential diagnosis between malignant and benign colonic neoplasms. This is one of the biggest clinical problems associated with large colonic lipomas. They can become symptomatic and may mimic clinical and endoscopic features of malignant colon tumors. This may cause symptoms similar to malignant colon tumors, such as constipation, diarrhea, abdominal pain, or rectal bleeding [5]. Benign polyps such as adenoma or malignant lesions such as adenocarcinoma are the main differential diagnoses in colonoscopy. Lipoma is the second most common benign colonic tumor following adenomatous polyps. They are usually solitary with varying sizes and may be sessile or pedunculated. Colonic lipoma is mostly diagnosed with colonoscopy as soft yellowish tumors or polyps that are described as cushion signs identified with pressure from biopsy forceps [3].

It is important to properly diagnose these lesions before choosing the treatment method. In cases where the diagnosis is uncertain or difficult, and a colonoscopic biopsy is noncontributory, further evaluation can be done using CT or endorectal ultrasonography (ERUS).

In the literature, the decision to resect lipomas and whether to choose the endoscopic or surgical technique remains a subject of controversy [7]. With advances in colonoscopy, endoscopic resection of colonic lipomas has been proven to be a safe and efficacious therapeutic method, especially for small lipomas. However, endoscopic removal of colonic lipomas larger than 2 cm is not widely used because of the risk of complications, including colonic perforation [8]. Indeed, as the majority of lipomas are submucosal, endoscopic removal carries a high risk of morbidity due to perforation, as its high-water content requires an enormous amount of heat to cut the lipoma [9]. However, some authors have reported that large sessile lesions can be removed without the risk of perforation. Kim et al. [10] have performed endoscopic removal of lipomas up to 3.8 cm in diameter, assisted by the injection of saline with or without epinephrine into the submucosa beneath the lesion, without any complications. This case is similar to that of our patient.

Different views regarding the endoscopic removal of large lipomas have been reported. Surgical treatment has been another option employed for large colonic lipomas due to the fear of complications (such as bleeding or perforation after endoscopic snare resection) [7]. Recently, laparoscopic procedures have been described as an alternative to conventional laparotomy for the resection of large colonic lipomas [8]. They have been shown to be associated with less postoperative pain, a shorter duration of ileus, and faster recovery [11]. Segmental colonic resection or hemicolectomy is recommended for complete removal of the lipoma.

The positive diagnosis of colonic lipoma is often made after a pathological study of the resected mass. Histologically, colonic lipomas are usually located in the submucosa. They may show prominent fibrosis or vasculature [9].

## Conclusions

Colonic lipomas are benign and usually diagnosed incidentally during colonoscopy. The diagnosis of a typical colonic lipoma is not very difficult. However, in cases of large lipomas, it would be important to distinguish between the lipoma and the malignant tumor. Proper radiological and colonoscopic evaluation is essential to avoid unnecessary wide surgical resection. Based on our findings in this report, we believe that large colonic lipomas can be safely removed by endoscopic resection, even though this can be technically challenging due to their vascular nature, large pedicle, and size. It is imperative to establish a proper preoperative diagnosis in order to choose the appropriate procedure.
